# Retrospective evaluation of correlation and agreement between two recovery scoring systems in horses

**DOI:** 10.1136/vr.104546

**Published:** 2017-12-01

**Authors:** Stefania Scarabelli, Eva Rioja

**Affiliations:** School of Veterinary Science, Philip Leverhulme Equine Hospital, University of Liverpool, Neston, UK

**Keywords:** anaesthesia, horses, recovery

Recovery is one of the most important and risky phases in equine anaesthesia.^1^Several recovery scoring systems (RSS) have been developed to evaluate risk factors associated with morbidity and mortality during this perioperative phase. A descriptive scale^2^ and a composite rating scale^3^ are commonly used in equine practice and have been reported as reliable methods for qualifying recovery from general anaesthesia in horses^4^; however, a truly validated and universally used RSS is not available at present, complicating the comparison between different studies in which different scales are used.

The aim of this study was to add information to the existing knowledge about recovery in horses, evaluating correlation and agreement between the two RSS used at the authors’ institution.

Anaesthetic records of horses undergoing general anaesthesia between January 2013 and November 2015 were retrospectively reviewed. During this period, two types of recovery scores were used: Y, modified from Young and Taylor,^2^ and D, modified from Donaldson and others.^3^ System Y (Table 1) is a simple descriptive scale that employs a scale from 1 to 5 to score recovery quality in its entirety; it assigns several descriptors to each score, and high numerical values indicate ‘worst’ recovery.

**TABLE 1:** System Y (modified from Young and Taylor^2^)

Score 1	Excellent: perfect, no ataxia; stands at first attempt as if rising from rest
Score 2	Good: stands after first or second attempt, some weakness/ataxia and may knuckle slightly after standing but not in danger of falling over
Score 3	Fair: stands after several attempts, crashes/ricochets around recovery box in excited/delirious manner often giving moments for concerns; moderate weakness/ataxia and may knuckle on fetlocks after standing; looks very wobbly and may teeter occasionally, but if falls, gets up again almost immediately; may sustain minor cuts/grazes but not requiring serious treatment intervention (not requiring suturing or further general anaesthesia to treat)
Score 4	Marginal: multiple attempts to stand, crashes/ricochets around recovery box in excited, almost manic manner giving rise to concern; very ataxic when finally standing and commonly falls down again; sustains injury requiring intervention, and which may compromise horse’s future/use
Score 5	Poor/unsuccessful: multiple attempts to stand, crashes/ricochets around recovery box in excited, manic manner perhaps until exhausted; may have multiple frantic attempts to stand between exhausted episodes; fails to stand because of postanaesthetic myopathy/neuropathy or through sustaining major/terminal injury (eg, long bone fracture, neck or skull-base fracture, dislocation)

System D (Table 2) is a composite scoring system in which eight phases of the recovery are scored and then the values are summated to obtain a total score that is then matched to an overall descriptive score developed to rank the recovery on a scale from 1 to 5 according to the quality of recovery, with the highest number of the descriptive score indicating the worst recovery, similar to system Y.^5^

**TABLE 2:** System D (modified from Donaldson and others and Valverde and others^3 5^)

Overall attitude	1–Calm 2–Calm/determined 3–Confused, dizzy 4–Frantic
Move to sternal	1–Smooth/methodical 2–Fighting mat, but controlled 3–Crashing, flopping over
Sternal phase	1–An organised pause 2–Non-existing 3–Multiple, with struggle
Moved to sternal	1–Methodical 2–An organised scramble 3–Used walls for support 4–Ricocheting off walls
Strength	1–Near full 2–Mildly rubbery 3–Dog sitting before standing 4–Repeated attempts due to weakness
Number of attempts to stand	Score = #
Balance and coordination	1–Solid 2–Moderate dancing 3–Reflex saves 4–Careening 5–Falls back down
Knuckling	1–None 2–Hindlimbs only 3–All four
Based on total score	1–Smooth, calm
	2–Calm but with some weakness, mild ataxia less than 10 minutes in duration
	3–As 2 but ataxia more than 10 minutes in duration
	4–Uncoordinated, with difficulties and ataxia, more than 3 attempts to stand
	5–Difficult recovery, unable to stand in the first 5 attempts; ataxia that can last for more than 20 minutes

All horses included in the study had unassisted recoveries. For each horse, recoveries were scored with both systems by the same person, whose identity was not specified upon the anaesthetic record but that was either a diplomate of the European or American College of Veterinary Anaesthesia and Analgesia, a resident in Veterinary Anaesthesia and Analgesia, or a final-year student.

Horses were included in the study if data about American Society of Anaesthesiologists (ASA) grade,^6^ type of surgery (emergency or elective) and both recovery scores were reported in the anaesthetic records. Demographic data of the horses were also recorded.

Data were analysed using commercially available statistical software (SPSS, V.22.0, 2013). Correlation and agreement were evaluated with Spearman’s coefficient and *k*-weighted, respectively. These analyses were performed in all the cases grouped together and after subdividing the horses into groups based on ASA category and type of procedure.

Eight hundred and twenty-three horses were included in the study; 473 were geldings, 301 mares and 49 stallions. The age range was 1–33 years, the median age was 10 and the interquartile range (IQR) was 8 years; the weight range was 53–892 kg, the median was 526 and the IQR was 144 kg. Several breeds were included in the study, with Thoroughbred being the most represented one (158). ASA I category included 284, ASA II 285, ASA III 157, ASA IV 88 and ASA V 9 horses. Five hundred and eleven horses underwent elective procedures, whereas 312 underwent emergency surgery, with exploratory laparotomy for colic surgery being the most represented procedure.

The Spearman’s correlation coefficient between the two systems was 0.88 (confidence interval 0.86 to 0.89; P<0.01); overall *k*-weighted was 0.65. When horses were divided into groups based on ASA category, *k*-weighted was 0.64 for ASA I, 0.69 for ASA II, 0.59 for ASA III, 0.63 for ASA IV and 0.82 for ASA V. When horses were divided into groups based on type of surgery, *k*-weighted was 0.68 for elective and 0.59 for emergency procedures.

In 27 per cent of cases, D score was higher than Y, whereas only in 2 per cent of cases Y score was higher than D. In 528 (64 per cent) horses, the two scoring systems gave the same score; in 282 (34 per cent) horses the score difference was only one point and in 13 (1.6 per cent) cases the difference was two points. Among these 13 cases, in 11 cases D and Y systems scored 5 and 3, respectively, and in two cases D system scored 4 and Y system scored 2. Most commonly, the disagreement between the two systems was for recovery scores 1–2 and 2–3.

Both Young and Taylor’s^2^ and Donaldson and others’^3^ systems have been used to score recoveries from general anaesthesia in clinical studies in horses, and they have been considered reliable^4^ and repeatable.^7^ Strong correlation between the two systems has already been reported^8^ and has been confirmed with our results; however, correlation does not reflect agreement, and therefore in our study *k*-weighted was calculated too. Substantial agreement (*k*=0.65) was reported between the two systems overall, but almost perfect agreement (*k*=0.82) was found only for horses belonging to the ASA V category, confirming what has been previously reported by Suthers and others.^7^

In our study, D system gave higher scores than Y system in 27 per cent of cases; this fact must be considered if multicentre studies are performed including facilities that use different RSS.

Clark-Price and others^9^ reported that subjective composite scales can lead different raters to different conclusions about the recovery status of the horse; ideally, an objective method should be developed in order to avoid inter-rater and intrarater variability, allowing comparison of studies performed at different centres.

The main limitation of our study is that people with different levels of experience were involved in scoring the recoveries. In fact, in our teaching hospital, the recovery score is often performed by students under the supervision of experienced equine anaesthetists (diplomates of the European or American College of Veterinary Anaesthesia and Analgesia) or anaesthesia residents, but the name of the person effectively scoring the recovery was not reported in the anaesthetic record; it was therefore not possible for the authors to record the level of experience of the observer or to include it in the analysis. However, there is evidence that the observer’s experience does not affect recovery quality scoring.^4 10^

## Conclusions

A very strong correlation between Y and D recovery scoring systems exists. Their agreement was overall substantial, but the D system will generally give higher scores than the Y system. This fact should be considered when comparing studies using these two systems to evaluate recovery quality or if a multicentre study is performed.
